# Laparoscopic subtotal cholecystectomy after percutaneous transhepatic gallbladder drainage for grade II or III acute cholecystitis

**DOI:** 10.1186/s12893-021-01387-w

**Published:** 2021-10-30

**Authors:** Masafumi Ie, Morihiro Katsura, Yukihiro Kanda, Takashi Kato, Kazuya Sunagawa, Hidemitsu Mototake

**Affiliations:** Department of General Surgery, Okinawa Chubu Hospital, 281 Miyazato, Uruma, Okinawa Japan

**Keywords:** Percutaneous transhepatic gallbladder drainage, Subtotal cholecystectomy, Safety, Feasibility

## Abstract

**Background:**

Severe adhesions and fibrosis between the posterior wall of the gallbladder and liver bed often render total cholecystectomy after percutaneous transhepatic gallbladder drainage (PTGBD) difficult, leading to high open conversion rates. Since the publication of Tokyo Guidelines 2018 (TG18), our policy has shifted from open conversion to subtotal cholecystectomy (SC) when total laparoscopic cholecystectomy for difficult cases of cholecystitis is not feasible. Recently, SC has been frequently applied as bailout surgery for complicated cholecystitis. Nonetheless, the efficacy and validity of laparoscopic SC after PTGBD remain unclear. This study aimed to evaluate the safety and feasibility of laparoscopic SC after PTGBD for grade II or III acute cholecystitis (AC) by comparing two periods of altered surgical strategies.

**Methods:**

This retrospective cohort study was conducted between January 2013 and December 2020. A total of 44 eligible patients with grade II or III AC were divided according to the time of cholecystitis onset into the pre-TG18 group (2013–2017, *n* = 17) and post-TG18 group (2018–2020, *n* = 27). Patients’ background demographics, surgical method, surgical results, and postoperative complications were compared.

**Results:**

The interval between PTGBD and surgery was significantly longer in the post-TG18 group than in the pre-TG18 group (15 [interquartile range: 9–42] days vs. 8 [4–11] days; *P* = 0.010). The frequency of laparoscopic cholecystectomy significantly increased from 52.9% in the pre-TG18 group to 88.9% in the post-TG18 group (*P* = 0.007), whereas the frequency of SC was 23.5% and 40.7%, respectively, which showed no statistically significant difference (*P* = 0.241). However, the rate of laparoscopic SC significantly increased from 0 to 90.9% among 15 SC cases, whereas the rate of open SC significantly plummeted from 100 to 9.1% (*P* = 0.001). Significant differences in the operative time, amount of intraoperative bleeding, and incidence of postoperative complications (wound infection and subhepatic abscess) were not observed. Mortality, bile leakage, and bile duct injury did not occur in either group.

**Conclusions:**

For grade II or III AC after PTGBD, aggressive adoption of SC increased the completion rate of laparoscopic surgery. Laparoscopic SC is a safe and feasible treatment option.

**Supplementary Information:**

The online version contains supplementary material available at 10.1186/s12893-021-01387-w.

## Background

The Tokyo Guidelines 2018 (TG18) recommend that percutaneous transhepatic gallbladder drainage (PTGBD) should be followed by elective/delayed cholecystectomy for moderate or severe acute cholecystitis (AC) in patients with a poor general condition, which does not improve with antimicrobial therapy or general supportive treatment [1]. Minimizing the invasiveness of cholecystectomy and limiting the perioperative complications are important issues because several patients undergoing PTGBD are elderly and/or have severe comorbidities [2]. However, severe fibrosis and adhesion render dissection difficult during laparoscopic cholecystectomy (LC) for AC after PTGBD, leading to reportedly high open conversion rates [3]. Furthermore, open conversion is not always safe and does not make total cholecystectomy easier for surgeons who lack considerable experience in performing open cholecystectomy [4]. According to the TG18, the decision to perform open conversion should be made after considering the surgeon’s skill and experience; subtotal cholecystectomy (SC) is an acceptable procedure if the risk of total cholecystectomy is high even after laparotomy [5].

In our institution, open conversion is selected for difficult cases of AC when total LC is not feasible. Since the publication of TG18, laparoscopic SC has been adopted instead of open conversion. Laparoscopic SC has been extensively used over recent years, and its effectiveness for complicated cholecystitis has been reported [6]. Nonetheless, the efficacy and feasibility of laparoscopic SC after PTGBD remain unclear. Therefore, this study aimed to evaluate the safety and feasibility of laparoscopic SC for grade II or III AC after PTGBD by comparing two periods of altered surgical strategies before and after the publication of TG18.

## Methods

We conducted a retrospective cohort study using data collected from a single public hospital in Okinawa, Japan (Okinawa Chubu Hospital) between January 2013 and December 2020. This study included 47 patients who underwent PTGBD for grade II or III AC, followed by cholecystectomy. Two patients who underwent additional cholecystectomy for other gastrointestinal malignancies and one patient with a preoperative diagnosis of cholecystocolonic fistula were excluded. All the data related to the present study were extracted from the medical records. The diagnosis of AC was made based on physical findings, ultrasound examination, and/or computed tomography. The severity (grade) of AC was determined according to the TG18 [7]. The surgeons decided on PTGBD for patients who were deemed to have poor tolerability of emergency surgery based on age, duration from the onset, and presence of comorbid diseases, as well as for patients whose symptoms did not improve with intravenous antimicrobial therapy. To clarify the differences in severity and general condition between the direct cholecystectomy group and the post-PTGBD cholecystectomy group, a supplementary comparison was conducted in the post-TG18 group. The 44 patients enrolled in this study were divided into two groups according to the time of cholecystitis onset: the pre-TG18 group (2013–2017, *n* = 17) and the post-TG18 group (2018–2020, *n* = 27). A comparative study on the clinical features, surgical methods, short-term postoperative outcomes, and postoperative complications between the two groups was conducted. This study was reported according to STROBE guidelines for observational studies, where appropriate [8].

### Medical treatment for acute cholecystitis

After the diagnosis of acute cholecystitis, antimicrobial therapy and fluid replacement therapy was started immediately. Cefmetazole, a second-generation cephem antibiotic, was administered as the primary antimicrobial therapy. If the results of the previous bile culture were available, the corresponding antibiotic agent was selected. In patients with septic shock, broad-spectrum antimicrobial agents such as imipenem/cilastatin or meropenem were administered, and intravenous noradrenaline was used to stabilize blood pressure.

### Procedures and management for PTGBD

After performing ultrasound-guided transhepatic gallbladder puncture with an 18-G needle, a 7- to 8-Fr pigtail catheter was placed in the gallbladder under fluoroscopy using a guidewire. Surgery was scheduled after PTGBD following stabilization of the patient’s general condition. If early cholecystectomy could not be performed, the gallbladder was imaged through the PTGBD tube. The PTGBD tube was removed if there was no obstruction of the cystic duct, and cholecystectomy was scheduled depending on the improvement in the general condition. PTGBD was continued until surgery if the cystic duct was obstructed and the tube was removed intraoperatively.

### Bailout surgery

The fundus-first technique was used if difficulties were encountered while removing the tissue surrounding the Hartmann pouch or if the cystic duct was dilated and anatomical identification was uncertain. The cystic duct was ligated with ENDOLOOP^®^ Ligature, or the stump of the gallbladder infundibulum was sutured and closed with 000 absorbent threads. SC was performed if the fibrosis and adhesion were severe and if the patient was assumed to be at risk for increased bleeding from the bare liver and damage to surrounding organs, such as the common bile duct and duodenum. The remaining gallbladder mucosa was cauterized with spray coagulation using monopolar electrocautery.

The timing of open conversion was decided by the attending surgeon. The inability to identify the cystic duct and gallbladder artery within 1 h and uncontrollable bleeding were factors that encouraged open conversion.

### Statistical analysis

Statistical analyses were performed using StatView version 5.0 (SAS Institute Inc., Cary, NC, USA). Descriptive analysis was performed to summarize the patients’ background demographics and practice patterns in both groups. Data were presented as median and interquartile range (IQR) or as number and percentage (%), as appropriate. Chi-squared test was used to compare the proportion of categorical variables, whereas the Mann–Whitney U test was employed to compare the medians of continuous variables. The clinical and laboratory parameters, time from onset to PTGBD, time from PTGBD to cholecystectomy, operative method, and outcomes were evaluated. To assess the differences in treatment strategies and surgical outcomes, we conducted a univariate analysis to compare the variables in the pre-TG18 and post-TG18 groups. Statistical analyses were two-sided, with a *P*-value of 0.05 being considered to be indicative of statistical significance.

## Results

Of the 545 patients diagnosed with acute cholecystitis between January 2013 and December 2020, 272 patients underwent cholecystectomy. Among them, PTGBD was inserted preoperatively in 47 cases (17.3%). Two patients who underwent additional cholecystectomy for other gastrointestinal malignancies and one patient of cholecystocolonic fistula were excluded. The comparison of the severity and general condition between the direct cholecystectomy group (n = 77) and the post-PTGBD cholecystectomy group (n = 27) for AC of grade II or III after TG18 were shown in Additional file 1: Table S1. The background of the post-PTGBD cholecystectomy group was significantly older, more severe, and had more comorbidities.

The clinical characteristics, time interval between cholecystitis onset and PTGBD placement, and time from PTGBD placement to cholecystectomy are summarized in Table 1. Significant differences were not observed between the two groups with respect to age, severity or grade of AC, systemic inflammatory response syndrome, white blood cell count on admission, and Charlson Comorbidity Index. There were significantly more men in the post-TG18 group (*P* = 0.042). The rate of American Society of Anesthesiologists physical status (ASA-PS) class I–II was lower in the post-TG18 group (70.6% vs. 48.1%), whereas the rate of ASA-PS class III was higher, albeit without any significant difference (29.4% vs. 51.9%, *P* = 0.143). The median interval between cholecystitis onset and PTGBD placement was 3 (IQR: 1–7) days in the pre-TG18 group and 2 (IQR: 1–3) days in the post-TG18 group; however, the difference between them was not statistically significant (*P* = 0.126). On the other hand, the median time from PTGBD placement to cholecystectomy was significantly prolonged from 8 (IQR: 4–11) days in the pre-TG18 group to 15 (IQR: 9–42) days in the post-TG18 group (*P* = 0.010).

**Table 1:** Clinical characteristics of the pre-TG18 group and post-TG18 group

Variable	Pre-TG18 group (2013–2017, *n* = 17)	Post-TG18 group (2018–2020, *n* = 27)	*P-*value
Age (years), median [IQR]	75 [63–84]	78 [68–87]	0.682
Sex Male	6 (35.3%)	18 (66.7%)	0.042*
Severity grade of AC			0.234
II	16 (94.1%)	22 (81.5%)	
III	1 (5.9%)	5 (18.5%)	
ASA-PS			0.143
I–II	12 (70.6%)	13 (48.1%)	
III or higher	5 (29.4%)	14 (51.9%)	
SIRS	10 (58.8%)	18 (66.7%)	0.599
WBC count on admission × 10^3^/μL, median [IQR]	13.1 [10.7–20.6]	14.9 [11.9–19.7]	0.736
CCI on admission	1 [0–2.5]	2 [1–3]	0.219
Comorbidity			
MI	5 (29.4%)	6 (22.2%)	0.592
CHF	1 (5.9%)	4 (14.8%)	0.363
Peripheral vascular disease	0	3 (11.1)	0.155
Cerebrovascular disease	3 (17.6%)	5 (18.5%)	0.942
Dementia	3 (17.6%)	6 (22.2%)	0.714
RA	0	2 (7.4%)	0.251
Diabetes	5 (29.4%)	8 (29.6%)	0.988
Hemiplegia	0	4 (14.8%)	0.096
CKD	0	2 (7.4%)	0.251
Solid tumor	1 (5.9%)	2 (7.4%)	0.845
Lymphoma	1 (5.9%)	0	0.202
Time from onset to PTGBD, days, median [IQR]	3 [1–7]	2 [1–3]	0.126
Time from PTGBD to cholecystectomy, days, median [IQR]	8 [4–11]	15 [9–42]	0.010*

*TG18* Tokyo Guidelines 2018, *AC* acute cholecystitis, *ASA-PS* American Society of Anesthesiologists physical status, *SIRS* systemic inflammatory response syndrome, *WBC* white blood cell, *CCI* Charlson Comorbidity Index, *MI* myocardial infarction, *CHF* congestive heart failure, *RA* rheumatoid arthritis, *CKD* chronic kidney disease, *PTGBD* percutaneous transhepatic gallbladder drainage, *IQR* interquartile range

Values are presented as *n* (%) or median [IQR, 25th and 75th percentile], as appropriate

*P values significant at (P < 0.05)

Table 2 shows the operative method and outcomes of the study sample. The frequency of LC significantly increased from 52.9% in the pre-TG18 group to 88.9% in the post-TG18 group (*P* = 0.007). The rate of SC among the 44 patients enrolled in this study was 23.5% and 40.7% in the pre- and post-TG18 groups, respectively; however, this showed no significant difference (*P* = 0.241). Furthermore, the frequency of laparoscopic SC significantly increased from 0 to 90.9% (*P* = 0.001) among 15 SC cases, whereas the rate of open SC significantly plummeted from 100 to 9.1% (*P* = 0.001). Significant differences in the operative time, amount of intraoperative bleeding, and incidence of postoperative complications (wound infection and subhepatic abscess) were not observed. Nevertheless, the median operative time (137 min vs. 127 min), blood loss (110 mL vs. 45 mL), and postoperative hospital stay (10 days vs. 8 days) all showed a trend toward reduction or shortening in the post-TG18 group. Mortality, bile leakage, and bile duct injury did not occur in either group.

**Table 2:** Surgical approach and outcomes

Variables	Pre-TG18 group (2013–2017, *n* = 17)	Post-TG18 group (2018–2020, *n* = 27)	*P-*value
Open surgery/laparoscopic surgery	8 (47.1%)/ 9 (52.9%)	3 (11.1%)/ 24 (88.9%)	0.007*
Open conversion rate	3 (33.3%)	3 (12.5%)	0.167
SC	4 (23.5%)	11 (40.7%)	0.241
Open SC/laparoscopic SC	4 (100.0%)/ 0	1 (9.1%)/ 10 (90.9%)	0.001*
Open conversion rate	0	3 (30.0%)	
Operative time, min [IQR]	137 [97–181]	125 [111–148]	0.373
Blood loss, mL [IQR]	110 [3–305]	45 [15–100]	0.353
Postoperative hospital stay, days [IQR]	10 [4–13]	8 [4–20]	0.515
Wound infection	1 (5.8%)	1 (3.7%)	0.736
Subhepatic abscess	1 (5.8%)	2 (7.4%)	0.845
Bile leakage	0	0	
Common bile duct injury	0	0	

*TG18* Tokyo Guidelines 2018, *SC* subtotal cholecystectomy, *IQR* interquartile range

Values are presented as *n* (%) or median (IQR, 25th and 75th percentile), as appropriate

*P values significant at (P < 0.05)

**Fig. 1:**
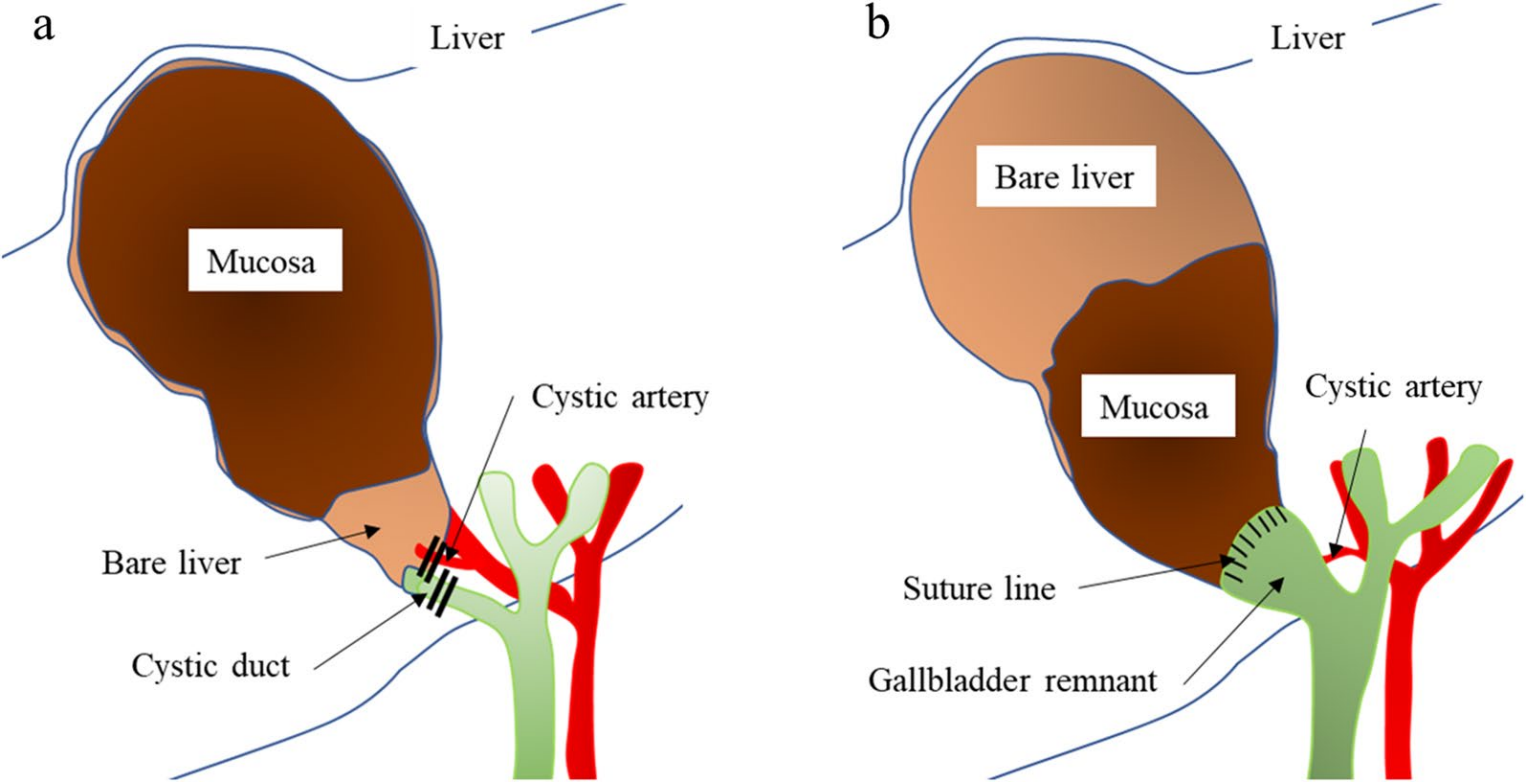
Types of subtotal cholecystectomy. **a** Hennemman type B [9, 21], which is characterized by the preservation of the posterior wall of the gall bladder with a closed remnant. **b** Strasberg reconstituting type A [10], in which the peritonealized wall of the gallbladder has been excised. The portion of the gallbladder adherent to the liver is retained or partially excised. The stump of the remnant of the gallbladder is closed with sutures

Table 3 summarizes the details of 15 SC cases. Henneman type B SC was performed in 13 patients (86.7%). This method entails the ligation and dissection of the cystic duct and gallbladder artery, and a part of the posterior wall of the gallbladder is retained (Fig. 1a) [9]. The remaining 2 patients (13.3%) underwent another SC procedure known as Strasberg reconstituting type A, a method of suturing and closing the stump of the gallbladder infundibulum that leaves a part of the posterior wall of the gallbladder (Fig. 1b) [10]. One case of Mirizzi syndrome was recorded.

**Table 3:** Details of subtotal cholecystectomy

Case no	Group	Mirizzi syndrome	Lap/open conversion	Fundus-first approach	Types of subtotal cholecystectomy		Wound infection	Subhepatic abscess
					Henneman classification	Strasberg classification		
1	Pre-TG18	−	Open	−	Type B		−	−
2	Pre-TG18	−	Open	−	Type B		−	−
3	Pre-TG18	−	Open	−	Type B		−	+
4	Pre-TG18	−	Open	−	Type B		−	−
5	Post-TG18	−	Conversion	+		Reconstituting type A	+	−
6	Post-TG18	−	Lap	+	Type B		−	−
7	Post-TG18	−	Lap	−	Type B		−	−
8	Post-TG18	−	Lap	−	Type B		−	−
9	Post-TG18	−	Conversion	+	Type B		−	−
10	Post-TG18	−	Lap	−	Type B		−	−
11	Post-TG18	−	Lap	−	Type B		−	−
12	Post-TG18	−	Lap	+	Type B		−	−
13	Post-TG18	+	Conversion	−		Reconstituting type A	−	+
14	Post-TG18	−	Open	−	Type B		−	−
15	Post-TG18	−	Lap	+	Type B		−	−

*TG18* Tokyo Guidelines 2018

## Discussion

The present study investigated patients with grade II or III AC who underwent PTGBD. This study revealed that the completion rate of laparoscopic surgery and the rate of laparoscopic SC were significantly higher in the post-TG18 group, which prolonged the period between PTGBD and cholecystectomy. Although major differences in surgical methods and treatment strategies were identified, they did not exert any detrimental effects on the surgical outcomes.

Technical difficulties and postoperative complications associated with severe fibrosis and gallbladder adhesion may be encountered in some patients who undergo LC after PTGBD [3, 11]. These issues are attributed to bleeding within the surgical field due to inflammation and the difficulty in anatomical identification caused by severe adhesion and scarring [12]. Some studies have investigated the relationship between the period between PTGBD and surgery and the postoperative complications to verify the optimal timing of cholecystectomy after PTGBD; however, no clear consensus has been established [13]. However, some studies conducted in recent years have supported delayed cholecystectomy [12–17]. Hye et al. reported a reduction in the operative time, postoperative hospitalization, and postoperative complications among patients who underwent cholecystectomy at 2 weeks or more after PTGBD placement [12]. Sakamoto et al. reported that the optimal time for performing cholecystectomy is between 7 and 26 days after PTGBD based on the mortality/morbidity data [14]. In a study conducted by Inoue et al. on 67 patients, the performance of cholecystectomy within 9 days after PTGBD was a risk factor for increased postoperative complications [15]. The advantages of delayed cholecystectomy include (1) the ability to perform surgery after the resolution of tissue and systemic inflammation as well as infection and (2) the stabilization of the patient’s general condition. In our study, the period from PTGBD to surgery was significantly prolonged in patients belonging to the post-TG18 group, who tended to be older, to have more severe cholecystitis, and to have poorer ASA-PS. These points suggest that patients in the post-TG18 group had a higher risk. In both periods, cholecystectomy was performed during the same hospitalization period as much as possible when the general condition was stabilized after PTGBD. This indicates that the post-TG18 group required a significantly longer time to stabilize their general condition because of the higher risk. Nevertheless, in the post-TG18 group, the median operative time, blood loss, and postoperative hospital stay all showed a trend toward reduction or shortening, and the postoperative complications did not increase. Our results suggest that delayed cholecystectomy may provide a beneficial effect on the surgical outcomes, even in more severe patients.

This study suggested that aggressive implementation of SC in the post-TG18 group was a factor that increased the completion rate of laparoscopic surgery. Horiuchi et al. reported that laparoscopic SC reduced the operative time, bleeding, postoperative hospitalization, and conversion rate in patients with AC with severe fibrotic adhesion in whom it was difficult to detach the posterior wall of the gallbladder [18]. Wee et al. studied SC in 168 cases and reported the absence of common bile duct injury and 30-day mortality [19]. The incidence of bile leakage after SC was reported to be 10–18% [9]. Compared to open cholecystectomy, SC was reported to result in greater postoperative bile leakage, but lower common bile duct injury, postoperative complications, reoperation, and mortality [20–22]. In this study, bile leakage did not occur in the 15 patients who underwent SC probably because ligation of the cystic duct or suture closure of the residual gallbladder stump was performed in all cases. Strasberg classified SC into the fenestrating and reconstituting types, whereas Henneman classified SC into four types (A, B, C, D) according to the difference in the management of the posterior wall and infundibulum of the gallbladder, respectively [9, 10]. Henneman examined the postoperative outcomes for each of the four types and reported that type B SC was not associated with complications such as laparotomy, bile leakage, reoperation, or postoperative endoscopic retrograde cholangiopancreatography [9]. In our study, 13 (86.7%) out of 15 patients underwent Henneman type B SC, which retained a part of the posterior wall of the gallbladder and allowed ligation and dissection of the cystic duct and artery (Fig. 1a). This finding indicates that inflammatory scarring and fibrosis between the posterior wall of the gallbladder and liver bed become more prominent as a result of gallbladder puncture and continuous drainage in patients with AC undergoing PTGBD, and the difficulty in dissecting the Calot triangle is not necessarily affected by PTGBD. Henneman type B SC was considered a safe and appropriate method for completing laparoscopic surgery even after PTGBD.

In our study, we showed that ligation and dissection of the cystic duct and artery were generally possible even after PTGBD. In this situation, most surgeons would attempt to perform total cholecystectomy. However, they may unexpectedly face difficulties in detaching the posterior wall of the gallbladder. Some surgeons may eventually adopt open conversion for total cholecystectomy, although the situation may not improve considerably. Adherence to total cholecystectomy can cause injury to the liver bed, increased bleeding, and slight bile leakage.

Regarding the generalization of our study results, it would be necessary to consider that the patient background of our cases. Our study population was slightly older than previous studies, limiting its generalizability [3, 12, 13, 15]. We believe adopting SC following PTGBD, rather than open conversion, may provide better surgical outcomes for older, high-risk patients with impaired physiological function.

There are several limitations to this study. First, this study was conducted with a small cohort of patients from a single institute. Second, the retrieval of data could have been incomplete or inaccurate owing to the retrospective study design. Third, there may have been some differences between individual surgeons regarding the indications for PTGBD. In other words, there was a situation in which surgery or PTGBD had to be selected, depending not only on the clinical status of the patient but also on the availability of human or physical resources at the institution.

## Conclusions

Our study suggested that delayed cholecystectomy after PTGBD could potentially improve the surgical outcomes but not increase the postoperative complications. Furthermore, SC increased the completion rate of laparoscopic surgery. Laparoscopic SC is a safe and feasible treatment option in post-PTGBD cholecystectomy for grade II or higher AC.

## Supplementary Information

**Additional file 1: Table S1** Comparison of clinical characteristics between the direct cholecystectomy group and the post-PTGBD cholecystectomy group after TG18. Table S1 demonstrated the background of the post-PTGBD cholecystectomy group was significantly older, more severe, and had more comorbidities.

## Data Availability

The datasets used and/or analyzed during the current study are available from the corresponding author on reasonable request.

## Abbreviations

AC: Acute cholecystitis
IQR: Interquartile range
LC: Laparoscopic cholecystectomy
PTGBD: Percutaneous transhepatic gallbladder drainage
SC: Subtotal cholecystectomy
TG18: Tokyo Guidelines 2018

## References

[1] Okamoto K, Suzuki K, Takada T, Strasberg SM, Asbun HJ, Endo I (2018). Tokyo guidelines 2018: flowchart for the management of acute cholecystitis. J Hepatobiliary Pancreat Sci.

[2] Abe K, Suzuki K, Yahagi M, Murata T, Sako H, Ishii Y (2019). The efficacy of PTGBD for acute cholecystitis based on the Tokyo Guidelines 2018. World J Surg.

[3] Chikamori F, Kuniyoshi N, Shibuya S, Takase Y (2002). Early scheduled laparoscopic cholecystectomy following percutaneous transhepatic gallbladder drainage for patients with acute cholecystitis. Surg Endosc Other Interv Tech.

[4] Lengyel BI, Azagury D, Varban O, Panizales MT, Steinberg J, Brooks DC (2012). Laparoscopic cholecystectomy after a quarter century: why do we still convert?. Surg Endosc.

[5] Wakabayashi G, Iwashita Y, Hibi T, Takada T, Strasberg SM, Asbun HJ (2018). Tokyo Guidelines 2018: surgical management of acute cholecystitis: safe steps in laparoscopic cholecystectomy for acute cholecystitis (with videos). J Hepatobiliary Pancreat Sci.

[6] Sabour AF, Matsushima K, Love BE, Alicuben ET, Schellenberg MA, Inaba K (2020). Nationwide trends in the use of subtotal cholecystectomy for acute cholecystitis. Surgery.

[7] Yokoe M, Hata J, Takada T, Strasberg SM, Asbun HJ, Wakabayashi G (2018). Tokyo Guidelines 2018: diagnostic criteria and severity grading of acute cholecystitis (with videos). J Hepatobiliary Pancreat Sci.

[8] von Elm E, Altman DG, Egger M, Pocock SJ, Gøtzsche PC, Vandenbroucke JP (2007). The Strengthening the Reporting of Observational Studies in Epidemiology (STROBE) statement: guidelines for reporting observational studies. Lancet.

[9] Henneman D, da Costa DW, Vrouenraets BC, van Wagensveld BA, Lagarde SM (2013). Laparoscopic partial cholecystectomy for the difficult gallbladder: a systematic review. Surg Endosc.

[10] Strasberg SM, Pucci MJ, Brunt LM, Deziel DJ (2016). Subtotal cholecystectomy-"fenestrating" vs “reconstituting” subtypes and the prevention of bile duct injury: definition of the optimal procedure in difficult operative conditions. J Am Coll Surg.

[11] Habib FA, Kolachalam RB, Khilnani R, Preventza O, Mittal VK (2001). Role of laparoscopic cholecystectomy in the management of gangrenous cholecystitis. Am J Surg.

[12] Jeon HW, Jung KU, Lee MY, Hong HP, Shin JH, Lee SR (2021). Surgical outcomes of percutaneous transhepatic gallbladder drainage in acute cholecystitis grade II patients according to time of surgery. Asian J Surg.

[13] Yamada K, Yamashita Y, Yamada T, Takeno S, Noritomi T (2015). Optimal timing for performing percutaneous transhepatic gallbladder drainage and subsequent cholecystectomy for better management of acute cholecystitis. J Hepatobiliary Pancreat Sci.

[14] Sakamoto T, Fujiogi M, Matsui H, Fushimi K, Yasunaga H (2020). Timing of cholecystectomy after percutaneous transhepatic gallbladder drainage for acute cholecystitis: a nationwide inpatient database study. HPB (Oxford).

[15] Inoue K, Ueno T, Nishina O, Douchi D, Shima K, Goto S (2017). Optimal timing of cholecystectomy after percutaneous gallbladder drainage for severe cholecystitis. BMC Gastroenterol.

[16] Kim HO, Ho Son B, Yoo CH, Ho SJ (2009). Impact of delayed laparoscopic cholecystectomy after percutaneous transhepatic gallbladder drainage for patients with complicated acute cholecystitis. Surg Laparosc Endosc Percutan Tech.

[17] Woodward SG, Rios-Diaz AJ, Zheng R, McPartland C, Tholey R, Tatarian T (2021). Finding the most favorable timing for cholecystectomy after percutaneous cholecystostomy tube placement: an analysis of institutional and national data. J Am Coll Surg.

[18] Horiuchi A, Watanabe Y, Doi T, Sato K, Yukumi S, Yoshida M (2008). Delayed laparoscopic subtotal cholecystectomy in acute cholecystitis with severe fibrotic adhesions. Surg Endosc Other Interv Tech.

[19] Tay WM, Toh YJ, Shelat VG, Huey CW, Junnarkar SP, Woon W (2020). Subtotal cholecystectomy: early and long-term outcomes. Surg Endosc.

[20] Elshaer M, Gravante G, Thomas K, Sorge R, Al-Hamali S, Ebdewi H (2015). Subtotal cholecystectomy for “difficult gallbladders”: systematic review and meta-analysis. JAMA Surg.

[21] Jara G, Rosciano J, Barrios W, Vegas L, Rodríguez O, Sánchez R (2017). Laparoscopic subtotal cholecystectomy: a surgical alternative to reduce complications in complex cases. Cir Esp.

[22] Matsumura T, Komatsu S, Komaya K, Ando K, Arikawa T, Ishiguro S (2018). Closure of the cystic duct orifice in laparoscopic subtotal cholecystectomy for severe cholecystitis. Asian J Endosc Surg.

