# Supplementary material for: Laparoscopic subtotal cholecystectomy after percutaneous transhepatic gallbladder drainage for grade II or III acute cholecystitis
Source: BMC Surg. 2021 Oct 30;21:386. doi: 10.1186/s12893-021-01387-w (PMC8557535; doi:10.1186/s12893-021-01387-w)
Supplement: Supplementary file 1 — Additional file 1: Table S1 Comparison of clinical characteristics between the direct cholecystectomy group and the post-PTGBD cholecystectomy group after TG18. Table S1 demonstrated the background of the post-PTGBD cholecystectomy group was significantly older, more severe, and had more comorbidities. [file 12893_2021_1387_MOESM1_ESM.docx]

**Supplementary Table 1** Comparison of clinical characteristics of the direct cholecystectomy group and the post-PTGBD cholecystectomy group after TG18

| **Variable** | **Direct cholecystectomy group (*n*=77)** | **Post-PTGBD cholecystectomy group (*n*=27)** | ***P-*value** |
| --- | --- | --- | --- |
| Age (years), median [IQR] | 65 [49–76] | 78 [68–87] | < 0.001* |
| Sex Male | 30 (39.0%) | 18 (66.7%) | 0.013* |
| Severity grade of AC |  |  | 0.001* |
| II | 76 (98.7%) | 22 (81.5%) |  |
| III | 1 (1.3%) | 5 (18.5%) |  |
| ASA-PS |  |  | 0.006* |
| I – II | 59 (76.6%) | 13 (48.1%) |  |
| III or higher | 18 (23.4%) | 14 (51.9%) |  |
| SIRS | 31 (40.3%) | 18 (66.7%) | 0.018* |
| CCI on admission, median [IQR] | 0 [0–2] | 2 [1 –3] | 0.001* |

*TG18* Tokyo Guidelines 2018, *AC* acute cholecystitis, *ASA-PS* American Society of Anesthesiologists physical status, *SIRS* systemic inflammatory response syndrome, *CCI* Charlson Comorbidity Index, *PTGBD* percutaneous transhepatic gallbladder drainage, *IQR* interquartile range

Values are presented as *n* (%) or median [IQR, 25^th^ and 75^th^ percentile], as appropriate

*p values significant at (p < 0.05)
